# Effective Delivery of PEGylated siRNA-Containing Lipoplexes to Extraperitoneal Tumours following Intraperitoneal Administration

**DOI:** 10.1155/2011/192562

**Published:** 2011-06-07

**Authors:** Akul Singhania, Sherry Y. Wu, Nigel A. J. McMillan

**Affiliations:** The University of Queensland Diamantina Institute, Princess Alexandra Hospital, The University of Queensland, Woolloongabba, QLD 4102, Australia

## Abstract

Intraperitoneal (i.p.) administration of small interfering RNA (siRNA) has, to date, shown promise in treating tumours located within the peritoneal cavity. The ability of these siRNA molecules to reach extraperitoneal tumours following i.p. administration is, however, yet to be investigated. Here, we examined the impact of PEGylation on the biodistribution of i.p. administered nucleic acids-containing lipoplexes. We showed that in contrast to non-PEGylated liposomes, PEGylated liposomes can deliver siRNA efficiently to extraperitoneal tumours following i.p. administration, resulting in a 45% reduction in tumour size when the oncogene-targeted siRNA was used. This difference was likely contributed by the decreased uptake of PEGylated lipoplexes in the first-pass organs, and, in particular, we observed a 10-fold decrease in the macrophage uptake of these particles compared to non-PEGylated counterparts. Overall, our results indicated the potential of using PEGylated liposomes to deliver siRNA for the treatment of i.p. localized cancer with coexisting extraperitoneal metastasis.

## 1. Introduction

Since its discovery, small interfering RNA (siRNA) has been widely investigated as a therapeutic agent to treat a wide range of human diseases, from cancer [1] to infectious [2] or neurodegenerative diseases [3]. For cancer therapy, it is typically administered locally (intracerebral/intratumoral injections) or systemically (intravenous injections) with the use of suitable delivery carriers, such as liposomes or polymers (reviewed in [4]). These carriers often carry a positive charge to interact efficiently with negatively charged siRNA thereby achieving effective entrapment or protection as well as permitting efficient cell entry. The promise of using siRNA to treat cancer has been recently demonstrated in a phase I clinical trial in which the repeated intravenous (i.v.) administrations of siRNA-containing cyclodextrin nanoparticles resulted in a significant downregulation of the targeted M2 subunit of ribonucleotide reductase (RRM2) protein in solid tumours [5].

Despite the success, the common adverse effects of repeated i.v. administration, such as phlebitis or loss of veins [6], along with the risk of embolism following injection [7], warrants research into alternative route of administration for cancer treatments. This is of particular interest for the administration of nucleic acids as the presence of both positively and negatively charged components in the formulation system often results in formation of aggregates in highly concentrated samples [8]. Given the intraperitoneal (i.p.) nature of several cancer types, including cancers of the digestive system, peritoneum, and ovaries [9], the i.p. route of administration presents as an attractive alternative. To date, three randomised phase III trials have already demonstrated the survival benefit of i.p. versus i.v. chemotherapy [10, 11] or when adjunct i.p. chemotherapy was used [12] for the treatment of advanced, low-volume ovarian cancer. Importantly, i.p., in contrast to i.v., delivery route permits large volume administration such that formulations can be prepared at dilute concentrations in order to avoid particle aggregation.

To date, several studies have described the successful i.p. delivery of DNA, antisense, or siRNA, using viral vectors [13, 14], nanoparticles [15], liposomes [1], or polymers [16]. Fewell and colleagues, for example, administered a polymer-complexed anticancer cytokine interleukin-12 gene intraperitoneally into a mouse model of disseminated ovarian cancer, resulting in a significant decrease in vascular endothelial growth factor in tumours and improved survival [16]. Similarly, Landen and colleagues demonstrated a 48–81% tumour reduction following intraperitoneal administration of liposome-entrapped siRNA and paclitaxel, a chemotherapeutic agent, in a mouse model of ovarian cancer [1]. Importantly, it was reported that the level of tumour reduction observed was comparable to concurrently-treated mice with paclitaxel and siRNA administered via i.v. route [1]. While these studies demonstrated the feasibility of delivering nucleic acids intraperitoneally for treatment of i.p. localised tumour, the potential of these i.p. administered nucleic acids to reach extraperitoneal tumours is yet to be confirmed. This is important as many types of cancers have the tendency to metastasize to extraperitoneal sites with the primary tumour located within the peritoneal cavity [17, 18]. 

To our knowledge, only one study, to date, has reported the potential of delivering siRNA to extraperitoneal tumours following i.p. administration using TransMessenger, a commercially available transfection reagent [19]. The level of tumour delivery as well as factors which could influence the practicality of this approach, however, is still yet to be investigated. The aim of this study was therefore to systematically investigate the potential of siRNA to reach extraperitoneal tumours following i.p. administration. The siRNA molecules was entrapped within either non-PEGylated or PEGylated liposomes and the biodistribution along with the level of uptake by i.p. macrophages were examined. The delivery efficacy of siRNA to extraperitoneal tumours was further assessed in mice bearing E6/7 oncogene-expressed extraperitoneal tumours. Overall, our results indicated the potential of PEGylated liposomes to deliver siRNA to extraperitoneal tumours following i.p. administration and could therefore be of use for the treatment of i.p. localized cancer with coexisting extraperitoneal metastasis.

## 2. Materials and Methods

### 2.1. Materials

Dioleoyl trimethylammonium propane (DOTAP) and cholesterol were purchased from Sigma (St Louis, Mo, USA). Polyethylene Glycol (PEG)_2000_-C16Ceramide conjugate was from Avanti Polar Lipids (Alabaster, Ala, USA) and dioleoylphosphatidylethanolamine (DOPE) was from Northern Lipids (Vancouver, Canada).

Oligodeoxynucleotides with sense sequence of 5′-GTCAGAAATAGAAACTGGTCATC-3′ and antisense sequence of 5′-GATGACCAGTTTCTATTTCTGAC-3′ were obtained from Invitrogen (Carlbad, Calif, USA). HPV16 E6/7-targeted siRNA (5′-GCAACAGUUACUGCGACGUUU-3′; 5′-ACGUCGCAGUAACUGUUGCUU-3′) and control siRNA (5′-UUAUGCCGAUCGCGUCACAUU-3′; 5′-UGUGACGCGAUCGGCAUAAUU-3′) were purchased from Sigma-Aldrich (St Louis, Mo, USA) in annealed form.

TC-1 cells (murine C57B/6 lung epithelial cells) were obtained from TC Wu [20] and were cultured in Dulbecco's Modified Eagle Media (DMEM; Invitrogen, Carlsbad, Calif, USA) supplemented with 10% heat-inactivated fetal bovine serum (FBS; Bovoge, Keilor East, Australia), 0.2% primocin (InvivoGen, San Diego, Calif, USA), and 2 mM L-glutamine (Invitrogen).

### 2.2. Liposome Formulations

Non-PEGylated DOTAP/Cholesterol (1 : 1 molar ratio) was prepared using the hydration of lipid film method as previously described [21]. Dried lipid film was hydrated using sterile 5% dextrose solution, and the final liposome concentration was 5 mM. After stabilizing at room temperature for two hours, small unilamellar liposomes were obtained via extrusion through 0.1  *μ*m pore size Nucleopore track-etched membranes using a Lipex extruder (Northern lipids, Vancouver, Canada). The resultant liposomes were then complexed with oligonucleotides at a nitrogen : phosphate (N : P) ratio of 4 : 1 with the final oligonucleotides concentration being 24 *μ*g /300 *μ*L.

PEGylated lipoplexes were formulated using the hydration of freeze-dried matrix (HFDM) technique [22]. DOTAP, cholesterol, DOPE and PEG_2000_-C16Ceramide with a molar ratio of 50 : 35 : 5 : 10 were used. Freeze-dried matrix was hydrated with sterile water and the final product contained 24  *μ*g oligonucleotides or siRNA in 300 *μ*L of isotonic sucrose solution.

The particle size and nucleic acids entrapment efficiency of both formulations were examined using the procedures described previously in Wu et al. [22].

### 2.3. Animal Studies

All animal experiments were approved by The University of Queensland Animal Ethics Committee, and 2-month-old female C57B/6 mice (Perth, ARC) were used in all studies.

#### 2.3.1. Peritoneal Macrophages Uptake Study

Two separate injections of PEGylated or non-PEGylated lipoplexes containing 36 *μ*g of FITC-conjugated oligonucleotides (450 *μ*L/dose) were administered intraperitoneally into each mouse. Injections were performed at the left and right lower quadrant of the peritoneal cavity, and all mice received a total dose of 72 *μ*g of liposome-entrapped oligonucleotides or the corresponding amount of empty liposomes. At 6 hours after i.p. administration, euthanized mice were injected intraperitoneally with 5 mL of PBS-Heparin-FCS (5% heat inactivated FCS and 4 U/mL Na heparin, DBL, Hospira Pty Ltd, Lake Forest, Ill, USA). Pooled peritoneal fluid of up to 3 mice was collected for each treatment group, and i.p. macrophages were identified via staining with APC-CD11b (BioLegend, San Diego, CA) and PE-F4/80 (BioLegend) antibodies. The percentages of FITC-positive cells were determined in CD11b- and F4/80-positive population using flow cytometry (BD-FACS Canto). 

#### 2.3.2. Biodistribution Study

Mice were inoculated with 1 × 10^6^ TC-1 cells suspended in 100 *μ*L PBS subcutaneously at the right abdominal side. On day 14 after the inoculation, non-PEGylated or PEGylated lipoplexes, which contained 72 *μ*g of Alexa Fluor 750-conjugated oligonucleotides, were injected intraperitoneally using the procedure described above. Empty liposome-treated mice were used as controls. At 24 hrs post injection, tumours and major first-pass internal organs (liver, kidneys, and spleen) were dissected and the fluorescence intensity of each organ was examined using excitation and emission wavelengths of 720 nm and 790 nm, respectively, in a Kodak In Vivo Imager. Results were subsequently analysed using the Kodak molecular imaging software.

#### 2.3.3. Tumour Growth Inhibition Study

Mice were injected with one million TC-1 cells, suspended in 100 *μ*L of sterile PBS, at the right abdominal side. On day 3, 7, and 10 following tumour cell inoculation, mice were treated intraperitoneally with isotonic sucrose solution (vehicle), empty liposomes, or PEGylated lipoplexes containing 72 *μ*g of either E6/7-targeted siRNA or control siRNA. Five mice were used per treatment group, and tumour size was monitored using callipers during the course of the experiment. All mice were sacrificed on day 13, and the tumour size of each mouse was recorded and analysed using the GraphPad Prism software (GraphPad software, La Jolla, CA). Student *t*-test was performed to assess the difference between treatment and control groups.

## 3. Results and Discussion

**Figure 1:**
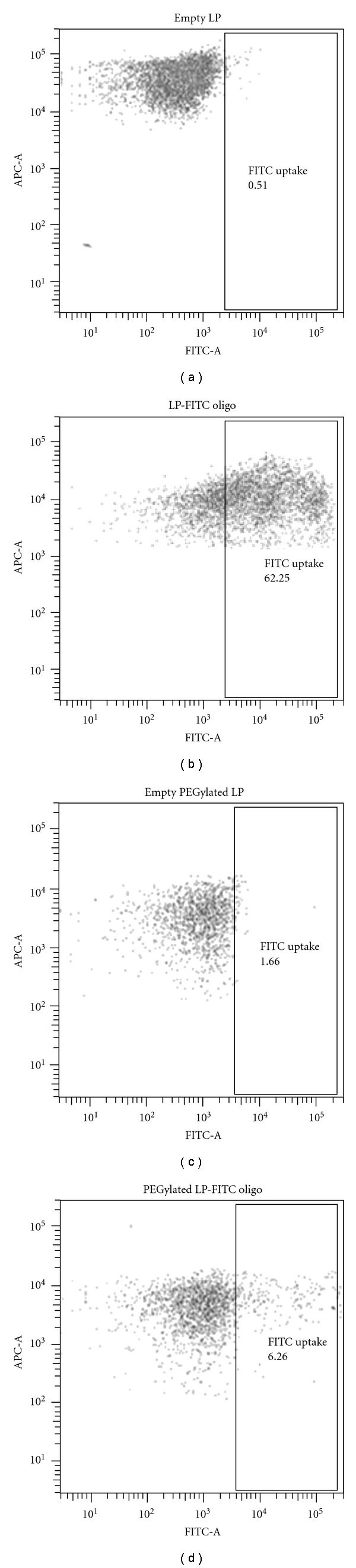
Uptake of FITC-oligonucleotides by intraperitoneal macrophages at 6 hrs after i.p. administration. Oligonucleotides were delivered using either (A) non-PEGylated or (B) PEGylated liposomes (LP), and macrophages were identified via APC-CD11b and PE-F4/80 staining. FACS plots are gated on APC-CD11b- and PE-F4/80-positive cells, and pooled peritoneal fluid of up to 3 mice was examined for each treatment group.

In order to examine the feasibility of delivering nucleic acids to extraperitoneal tumours following i.p. administration, we first sought to evaluate the level of uptake of oligonucleotides-containing lipoplexes by i.p. macrophages as they serve as our body's first line of defence by efficiently engulfing foreign particles. It was anticipated that a strong uptake of these particles by macrophages present in the peritoneal cavity would likely to significantly hinder their delivery to tumours, similar to what has been established with i.v. route of administration (reviewed in [23]). To address this, we formulated PEGylated lipoplexes in which the presence of PEG polymer on the particle surface would aid in their escape from the immune surveillance [24]. These particles were formulated using the hydration of freeze-dried matrix technique, resulting in final particles with an average size of 180–200 nm and an entrapment efficiency of >90% [22]. Similar particle size and entrapment efficiency were obtained for non-PEGylated lipoplexes. Oligonucleotides incorporated in these lipoplexes were FITC-labelled such that the level of uptake of these particles by i.p. macrophages could be easily assessed. Importantly, contrary to the study performed by Niu and colleagues where only 12 *μ*L of siRNA-containing complexes was administered intraperitoneally into each mouse [19], we administered diluted lipoplexes in a large volume to ensure even distribution of the particles within the peritoneal cavity and to reduce the potential formation of aggregates. This high-volume administration of diluted samples has been previously reported to result in reduced clearance leading to superior efficacy [25–29]. At 6 hrs after i.p. administration of lipoplexes, i.p. macrophages were isolated and identified via CD11b and F4/80 antibodies staining. Based on the FITC fluorescence signal detected in these macrophages, it was estimated that there was a 10-fold decrease in the level of uptake by macrophages for PEGylated lipoplexes compared to non-PEGylated ones (6.3% versus 62.3%, Figure 1).

**Figure 2:**
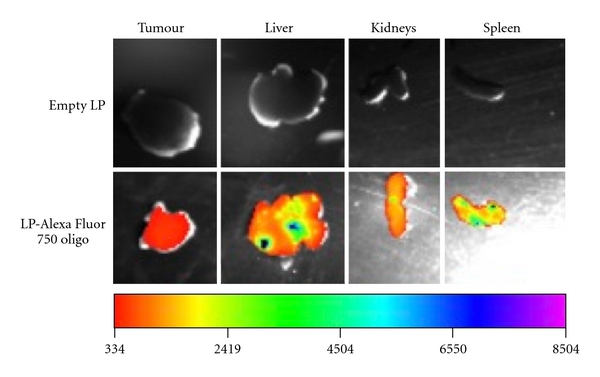
Biodistribution of non-PEGylated liposome-entrapped Alexa Fluor 750-conjugated oligonucleotides in tumours and major first-pass organs at 24 hrs following intraperitoneal (i.p.) administration in mice.

**Figure 3:**
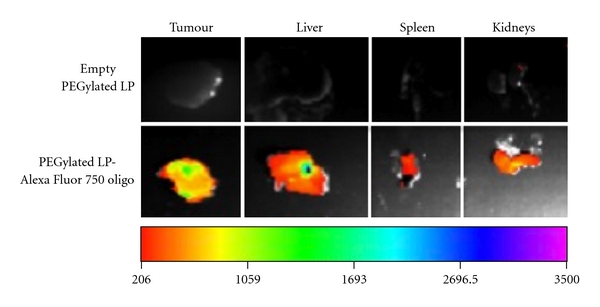
Biodistribution of PEGylated liposome-entrapped Alexa Fluor 750-conjugated oligonucleotides in tumours and major first-pass organs at 24 hrs following intraperitoneal (i.p.) administration in mice.

To examine whether this difference in macrophage uptake alters the biodistribution and the delivery efficiency of these particles to extraperitoneal tumours, we next labelled oligonucleotides with an infra red fluorescent dye, Alexa Fluor 750. The biodistribution of both PEGylated and non-PEGylated lipoplexes was subsequently examined in mice bearing extraperitoneal E6/7-expressing tumours. At 24 hrs after administration, it was found that the non-PEGylated lipoplexes accumulated mainly in the liver, spleen, and kidneys while very little tumour accumulation of these particles was observed (7.5% of total dose remained, Figure 2 and Table 1). In contrast, while a considerable amount of oligonucleotides still accumulated in first-pass organs, as expected [1, 29], the delivery of PEGylated lipoplexes to the extraperitoneal tumour was evident (Figure 3). This level of tumour localisation of PEGylated lipoplexes was found to be significantly more than that achieved by non-PEGylated ones (65.9% versus 7.5%, Figures 2, 3, and Table 1) and is consistent with what we have previously observed with i.v. route of administration of these particles [8]. Minimal delivery of either non-PEGylated or PEGylated lipoplexes was observed in the lungs (data not shown).

It must be noted that the PEGylated lipoplexes exhibited a significant decrease in spleen uptake compared to non-PEGylated lipoplexes following i.p. administration (7.8% versus 30%, Table 1). This could be contributed by the decreased uptake of these particles by i.p macrophages as it has been shown that intraperitoneal macrophages typically travel through the subcapsular sinus of parathymic lymph nodes and eventually reside in the parenchyma of the liver and spleen [30]. It is therefore likely that, following escape from first-pass organs, PEGylated lipoplexes were absorbed into subdiaphragmatic lymphatics at a much higher rate compared to non-PEGylated particles prior to entering the general circulation, which in turn contributed to their enhanced tumour localisation [6]. In addition, following absorption via mesenteric lymphatics, the slight reduction in liver uptake observed for PEGylated lipoplexes could also have contributed to the increased drainage of the administrative material into the portal veins [31] (Table 1).It is therefore anticipated that the PEGylated lipoplexes reached the extraperitoneal tumours via a combination of portal and lymphatics pathways. In contrast, diffusion across the peritoneum is less likely to be the route of transport for these particles to extraperitoneal sites as previous reports have shown that the largest pore size in the peritoneum is less than 40 nm [32, 33], which is significantly smaller than the size of our lipoplexes.

**Figure 4:**
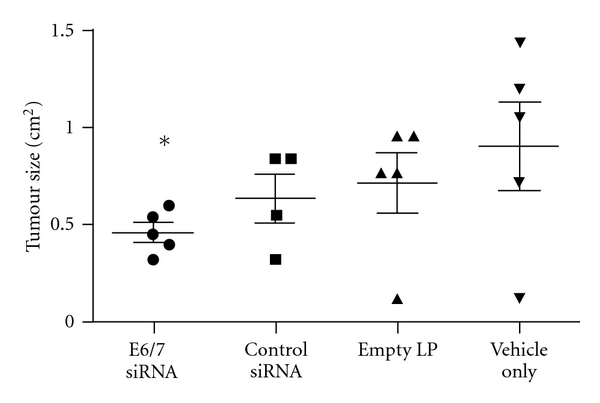
Inhibition of extraperitoneal TC-1 tumour growth by i.p. delivered siE6/7-containing PEGylated lipoplexes in mice. Mice received siRNA treatments on days 3, 7, and 10 following tumour cell inoculatios and tumour size was measured using callipers on day 13. All siRNAs were delivered using PEGylated liposomes (LP). Five mice were used per treatment group, and the error bars represent standard deviations. *P < .05, significantly different from the vehicle-only treatment group.

Having established the ability of PEGylated liposomes in delivering oligonucleotides to extraperitoneal tumours following i.p. administration, we next performed an efficacy study examining the antitumour effect of E6/7 siRNA when they are delivered intraperitoneally using these PEGylated lipoplexes. E6/7 was chosen as the target oncogene as it is exclusively present in tumour cells of cervical origin and its presence is absolutely essential for the proliferation of TC-1 cells used in this study [20]. Naked E6/7 siRNA, however, would not be able to be taken up by tumour cells readily following systemic administration [8].Effective tumour delivery of these E6/7-targeted siRNAs using an appropriate carrier was therefore essential in order to achieve a significant reduction in tumour growth rate, as we have previously demonstrated with i.v. route of administration [8]. Indeed, a 45% decrease in average tumour size was observed in mice treated with 3 doses of i.p. administered PEGylated siE6/7-containing lipoplexes when compared to vehicle-only-treated mice (Figure 4, *P* < .05). While similar level of tumour size reduction was reported to be achieved with a much more frequent administrations of a much lower dose/volume (2 *μ*g/12 *μ*L/dose, 12 injections) of siRNA in another study [19], we have found that the administration of such a low volume of lipoplexes into the peritoneal cavity is unlikely to yield a consistent level of delivery to extraperitoneal tumours (data not shown). It must be noted, however, that a slight reduction in tumour size was also observed in nontargeted siRNA or empty liposome treated groups compared to vehicle-only controls, although the level of reduction was not statistically significant (Figure 4). It is speculated at this time that this nonspecific reduction in tumour size observed could have been contributed by the mice's intrinsic response to repeated administrations of large volume/dose of liposomes. It remains to be investigated in the future as to whether enhanced tolerability and less nonspecific effects could occur when the dose was infusing slowly into mice without losing the efficacy [6]. Despite this, our results clearly indicated the promise of PEGylated liposomes in delivering siRNA to extraperitoneal tumours following i.p. administration.

## 4. Conclusions

To our knowledge, this is the first paper which systematically investigates the feasibility of delivering siRNA to extraperitoneal tumours following i.p. administration. We showed that, in contrast to non-PEGylated liposomes, PEGylated liposomes were able to facilitate the escape of siRNA from first-pass organs and deliver siRNA efficiently to extraperitoneal tumours after i.p injections. With the incorporation of E6/7-targeted siRNA, significant antitumour effect was observed in mice bearing extraperitoneal TC-1 tumours. Given the inconvenience of repeated i.v. administration along with the ease of aggregate formation for siRNA-containing formulations, our findings offer an attractive alternative for the treatment of cancers of peritoneal origin with the presence of extraperitoneal metastasis. 

## Figures and Tables

**Table 1:** Quantitative analysis of the distribution of Alexa Fluor 750-conjugated oligonucleotides in tumours and major first-pass organs at 24 hrs following intraperitoneal administration in mice. Oligonucleotides were delivered using either non-PEGylated or PEGylated liposomes (LP). The fluorescent images are presented in Figures 2 and 3.

	Average percentage of the total fluorescence intensity detected			
	Tumour	Liver	Kidney	Spleen
Non-PEGylated LP	7.5%	45.7%	15.7%	30.0%
PEGylated LP	65.9%	20.6%	7.8%	5.7%
